# Characterization of the biofilm forming ability of S*taphylococcus pseudintermedius* from dogs

**DOI:** 10.1186/1746-6148-9-93

**Published:** 2013-05-03

**Authors:** Ameet Singh, Meagan Walker, Joyce Rousseau, Jeffrey Scott Weese

**Affiliations:** 1 Department of Clinical Studies, Ontario Veterinary College, University of Guelph, Guelph, ON N1G 2W1, Canada; 2 Department of Pathobiology, Ontario Veterinary College, University of Guelph, Guelph, ON N1G 2W1, Canada

**Keywords:** Biofilm, *Staphylococcus pseudintermedius*, Microtitre plate assay, *ica*A, *ica*D

## Abstract

**Background:**

*Staphylococcus pseudintermedius* is an opportunistic pathogen of dogs and has emerged as a leading cause of skin, wound and surgical site infections worldwide. Methicillin resistance is common and clinical infections as a result of methicillin-resistant *S. pseudintermedius* (MRSP) pose a clinical challenge. In other staphylococci, biofilm formation has been shown to be a virulence factor for infection, however, it has received little attention in *S. pseudintermedius.* The objectives of this study were to evaluate the biofilm forming ability of clinical isolates of *S. pseudintermedius* obtained from dogs using phenotypic and genotypic techniques.

**Results:**

96% (136/140) of *S. pseudintermedius* isolates were classified as strong or moderate biofilm producers, with the majority of isolates being able to produce biofilm. There was no difference in biofilm formation between MRSP and MSSP (p=0.8), amongst isolates from clinical infections compared with isolates obtained from colonized dogs (p=0.08), and between isolates from sequence type (ST) 71 and ST 68 (P=0.09). *ica*A was detected in 77.9% (109/140) of isolates and *icaD* was detected in 75.7% (106/140) of isolates. Scanning electron microscopic evaluation of *S. pseudintermedius* biofilm production revealed aggregates of cocci and irregularly produced extracellular polymeric matrix.

**Conclusion:**

The majority of *S. pseudintermedius* isolates evaluated in this study were able to produce biofilm and this may be an important virulence factor in the rapid emergence of this bacterium in veterinary hospitals worldwide. Further study into the mechanisms of biofilm formation by *S. pseudintermedius* is warranted.

## Background

Surgical site infections (SSI) are an inherent risk of any surgical procedure and are reported as a complication in 0.8% to 18.1% of operations depending on wound classification [1-4]. The consequences of SSI in veterinary medicine include patient morbidity/mortality, prolonged hospitalization, increased treatment cost, frustration and grief of pet owners and caregivers alike. In veterinary surgical patients, SSI are becoming complicated by the emergence of multi-drug resistant bacteria as these infections are challenging to treat due to their resistance to many of the commonly used antimicrobials [5,6]. Of particular concern in dogs and cats has been the rapid emergence of methicillin-resistant *Staphylococcus pseudintermedius* (MRSP) [6]. This multidrug resistant opportunistic pathogen has spread rapidly and widely in recent years [6-8], and is the most common cause of SSI in some veterinary facilities [9].

Implant-related SSI are a serious complication and frequently result in implant removal since routine antimicrobial administration is rarely effective [10-13]. Implant removal results in additional patient morbidity, increased treatment costs, and, in some cases, may require re-application of a surgical implant [10-13]. It has been well documented that implant-related SSI in humans are complicated by the presence of bacterial biofilms [14,15]. A bacterial biofilm is a complex, sessile community of bacteria embedded within a self-produced matrix of carbohydrates, proteins and DNA (extracellular polymeric substance, EPS) [16-19]. Within a biofilm, bacteria have markedly altered metabolism, enhanced cell-to-cell communication, and are able to evade the host immune response and the effects of antimicrobials through their isolated metabolism along with physical and chemical protection of the biofilm matrix [17-19]. Following placement of an implant, it is rapidly coated with a host-derived, protein-based conditioning film, which contains receptors that allow for bacterial attachment initiating the process for biofilm formation [17-19]. The ability of bacteria to form a biofilm has been shown to be a leading cause of persistent SSI and, thus, the presence of a biofilm can greatly impact the ability to treat an SSI [17-19].

Biofilm formation is now recognized as an important virulence factor in several *Staphylococcus* spp. [20,21]. The ability to form a biofilm is likely variable between bacterial species, and the biofilm forming ability of *S. pseudintermedius* has not been fully characterized. One small report involving 23 MRSP isolates from dogs in Norway revealed that all isolates were biofilm producers with isolates belonging to sequence type (ST) 71 producing significantly more biofilm compared with other STs [22]. Another report revealed that clarithromycin was ineffective in eradicating MRSP biofilm at therapeutic doses [23]. In that study, all 20 MRSP isolates evaluated formed biofilm [23]. As MRSP has now become the leading cause of SSI in veterinary medicine, characterizing its biofilm forming ability will provide further insight into a rapidly emerging clinical dilemma.

Once initial attachment to a biomaterial occurs, a key step in biofilm formation is secretion of EPS [19]. In staphylococci, this extracellular “slime” component, also termed polysaccharide intercellular adhesion (PIA), is encoded, at least in part, by the *ica* operon [24,25]. In *S. epidermidis,* it has been shown that co-expression of *ica*A and *ica*D leads to an increase in the activity of PIA [25]. However, *ica*–independent biofilm formation has been reported in staphylococci [26] and the role of the *ica* operon in the biofilm forming ability of *S. pseudintermedius* is unknown.

The objectives of this study were to evaluate the biofilm forming ability of clinical isolates of *S. pseudintermedius* obtained from dogs using a quantitative microtitre plate assay (MPA), to compare biofilm formation amongst strains, and to determine the presence and impact of biofilm-associated genes (*ica*A and *ica*D).

## Methods

### Isolates

One-hundred and twenty one MRSP and 19 methicillin-susceptible *S. pseudintermedius* (MSSP) obtained from dogs were used. Isolates were obtained from a convenience sample of isolates from clinical infection or colonization in dogs from Canada and the United States. The isolates were collected from 2005 to 2012 from clinical cases and surveillance studies. The isolates were stored at −80°C in Cryostor beads (Innovatek Medical, Delta, BC).

### Phenotypic characterization of biofilm formation

Biofilm formation was evaluated using a quantitative spectrophotometric microtitre plate assay (MPA) as previously described [27]. Briefly, isolates were sub-cultured onto blood agar and pure 24hr growth used. Each isolate was suspended in 5.0 ml of tryptic soy broth (TSB) supplemented with 1% glucose to achieve a turbidity equivalent to a 0.5 McFarland-standard (~10^8^ CFU/ml). A 200 μl bacterial suspension was inoculated into a 96-well microtitre plate in triplicate and incubated overnight for 24 hr at 35°C without shaking to allow biofilm formation. Following incubation, the contents of the wells were discarded and each well was washed three times with sterile phosphate buffered saline (PBS) (pH 7.2) to remove non-adherent (planktonic) cells while carefully ensuring the integrity of formed biofilms was maintained. The adhered bacteria (biofilm) were heat fixed for 60 min at 60°C. Adhered cells were dyed with 150 μl of 0.1% (w/v) crystal violet for 15 min at 22°C and air dried. Following re-solubilization with 95% ethanol, optical density (OD) of each well was measured at 570 nm. Median OD_570_ of the triplicates of the negative control (TSB only) was subtracted from the median OD_570_ of triplicates of the samples. Based on the report by Stepanovich et al. [27], isolates were classified as strong, moderate, weak or zero biofilm producers based on their OD_570_ (4× ODc < OD_570_ = strong biofilm producer, 2× ODc < OD_570_ ≤ 4× ODc = moderate biofilm producer, ODc < OD_570_ ≤ 2× ODc = weak biofilm producer, OD_570_ ≤ ODc = no biofilm producer (ODcutoff (ODc) = average OD_570_ of negative control + (3× standard deviation of negative control)).

#### DNA extraction

Isolates were grown overnight on Colombia Blood agar and incubated at 35°C. A sample of a single colony (~10 μl) was re-suspended in 1 ml sterile water. The suspension was centrifuged at 12,000 rpm for 2 minutes. The supernatant was discarded and 200 μl of BIO-RAD InstaGene Matrix (Bio-Rad Laboratories, Montreal, Canada) was added to the pellet. The suspension was vortexed for 10 seconds and then incubated at 56°C for 30 minutes in a water bath. The suspension was then vortexed for 10 seconds and heated in a block heater for 8 minutes at 100°C. After heating, the suspension was centrifuged at 12,000 rpm for 2 minutes. The supernatant was then transferred to clean 200 μl PCR tubes. The extract was stored at −20°C.

### Real-time PCR detection of icaA and icaD genes

Real time polymerase chain reaction (RT-PCR) was performed in a DNA thermal cycler (CFX96 Real-Time system Thermocycler, Bio-Rad Laboratories Ltd, Montreal, Canada) and used to detect *ica*A and *icaD* in all isolates [28,29]. Gene sequences for *ica*A and *ica*D in *Staphylococcus pseudintermedius* were obtained from the National Centre for Biotechnology Information GenBank database (accession numbers: NC_014925.1 and NC_017568.1, respectively). The primer sequences used for RT-PCR for *ica*A were forward: 5’- TTGCCCACCTTGTGCCCACC-3’ and reverse: 5’- TGAGGCTGTAGGGCGTTGGGA-3’ and for *ica*D were forward: 5’- AGACGACACACCCTATGGCTATGAA-3’ and reverse: 5’- ACGTATTAGCGCACATTCGGTGTTA-3’. Reactions were carried out separately for *ica*A and *ica*D. The PCR reaction volume was 20 μl and contained 10 μl RT-PCR super mix (Bio-Rad SSoFast EvaGreen supermix, Bio-Rad Laboratories Ltd, Montreal, Canada) 0.5 μl MgCl_2_ (2.5 mM), 1 μl forward primer, 1 μl reverse primer, 5.5 μl sterile water, 2 μl bacterial DNA. Thermal cycling conditions were: an initial denaturation at 95° for 3 min, followed by 40 cycles of 10 seconds of denaturation at 95°, 10 seconds of annealing at 60°C, 30 seconds of extension at 72°. After the amplification cycles were complete, a melting curve analysis followed by ramping from 65°C to 95°C for 5 seconds. Samples with a crossing point (Ct <38) and a single melting peak consistent with the positive control were considered positive. Positive and negative controls were included with all runs. Representative PCR products of both *ica*A and *ica*D were sequenced to confirm identity of peaks in RT-PCR reactions.

### Biofilm structure evaluation by scanning electron microscopy (SEM)

Overnight culture of a strong biofilm producing MRSP isolate was inoculated with TSB + 1% glucose. A 316L stainless-steel 20 mm orthopaedic bone screw (Veterinary Orthopedic Implants, St. Augustine, FL, USA) was added to 5 ml of a 0.5 McFarland standard suspension and incubated for 24 hrs aerobically at 35°C. Following incubation, the screw was washed with PBS and fixed at 22°C with 2.5% glutaraldehyde until time of SEM imaging. The day prior to image acquisition, the screw was post-fixed with 1% osmium tetroxide for 30 min at 22°C, washed in Sorensen’s phosphate buffer twice for 15 min, dehydrated through an ethanol series (50%, 70%, 80%, 90%, 99.5% for 15 min each), critical point dried and sputter coated with gold. The screw was then imaged using a Hitachi S-570 SEM. Images were subjectively evaluated for adherent cells and extracellular matrix.

### MRSP characterization

MRSP isolates were characterized by sequence analysis of the *mec*-associated direct repeat unit (*dru* typing) [30]. The Dru repeats and types were assigned by the Dru-typing.org database (http://www.dru-typing.org/search.php). A minimum spanning tree was generated using BioNumerics v6.6 (Applied Maths, Austin, Texas, USA) and the TRST plugin. Distance intervals were created using a bin distance of 1.0%. Dru types separated by an MST distance of <2 (>98.5% similarity) were considered closely related and assigned to the same cluster [31]. The root node was assigned to the sequence type with the greatest number of isolates.

### Statistical analysis

Using commercially available software (JMP statistical discovery, Cary, NC, USA), descriptive statistics were performed and Student’s *t*-test used for continuous outcome data while Fisher’s exact and chi-square tests were used for categorical comparisons. Logistic regression analysis was also performed. A p value of <0.05 was considered significant.

## Results

**Figure 1:**
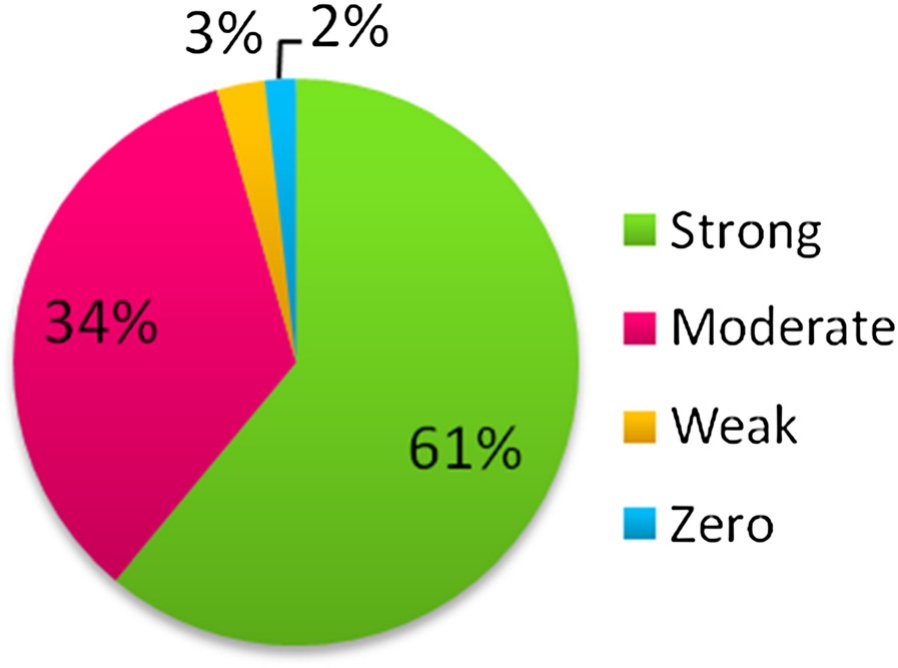
**Classification of *S. pseudintermedius *isolates based on biofilm-forming ability.**

Ninety-six percent (136/140) of *S. pseudintermedius* isolates were classified as strong or moderate biofilm producers, with the majority of isolates being able to produce biofilm to some degree (Figure 1). No difference in biofilm formation between MRSP and MSSP (p=0.8) was noted (Table 1). Ninety-three percent (132/140) of isolates belonged to two dru clusters, rooted by dt9a (n=58, 48%) and dt11a (n=55,45%) [32]. These correspond to the two major MRSP clonal complexes, sequence type (ST) 71 and ST 68, respectively. No significant difference in biofilm formation between these two clonal complexes (P=0.09) was observed (Table 1). Biofilm production was not significantly different amongst isolates from clinical infections compared with isolates obtained from colonized dogs (p=0.08) (Table 1).

**Table 1:** **Mean OD**_**570 **_**+/− standard deviation (SD) of various categories of *Scxx pseudintermedius *isolates evaluated**

**Categorization (n)**	**Mean OD**_**570 **_**+/− SD**
MRSP (121)	0.75 +/− 0.57
MSSP (19)	0.74 +/− 0.41
Infection (54)	0.80 +/− 0.58
Colonization (86)	0.65 +/− 0.48
ST 68 (55)	0.79 +/− 0.64
ST 71 (58)	0.62 +/−0.38

*ica*A was detected in 109/140 (77.9%) of isolates (MRSP - 94/121 and MSSP - 15/19). No association between the presence of *ica*A and OD_570_ value (P=0.77), being a strong (P=0.08) or strong/moderate biofilm producer (P=0.15), and between MRSP and MSSP isolates (P=1.0) were detected.

*icaD* was detected in 106/140 (75.7%) of isolates (MRSP - 93/121 and MSSP - 13/19). No association between the presence of *ica*D and OD_570_ value (P=0.47), being a strong (P=0.97) or strong/moderate biofilm producer (P=0.26), and between MRSP and MSSP isolates (P=0.40) were detected.

**Figure 2:**
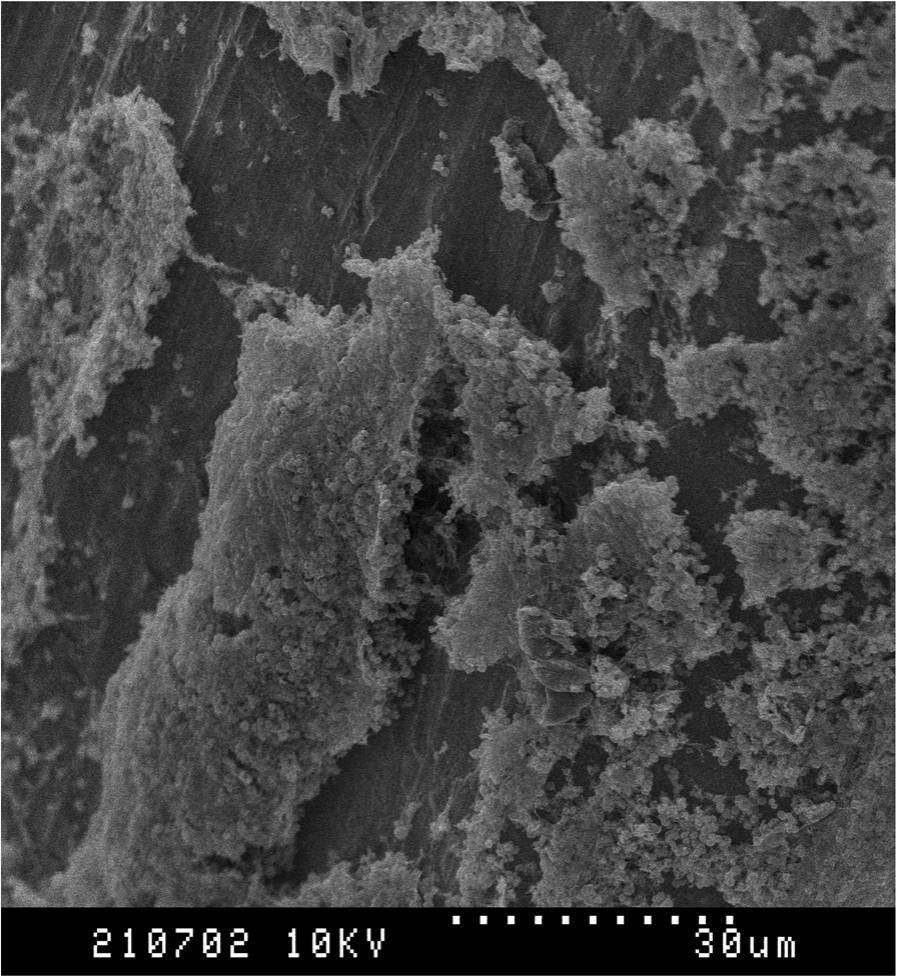
**SEM Images of MRSP biofilm formation on 316L stainless steel orthopedic bone screws.** Dotted line = 30 um. Large aggregates of cocci and irregularly produced extracellular polymeric substance are apparent.

**Figure 3:**
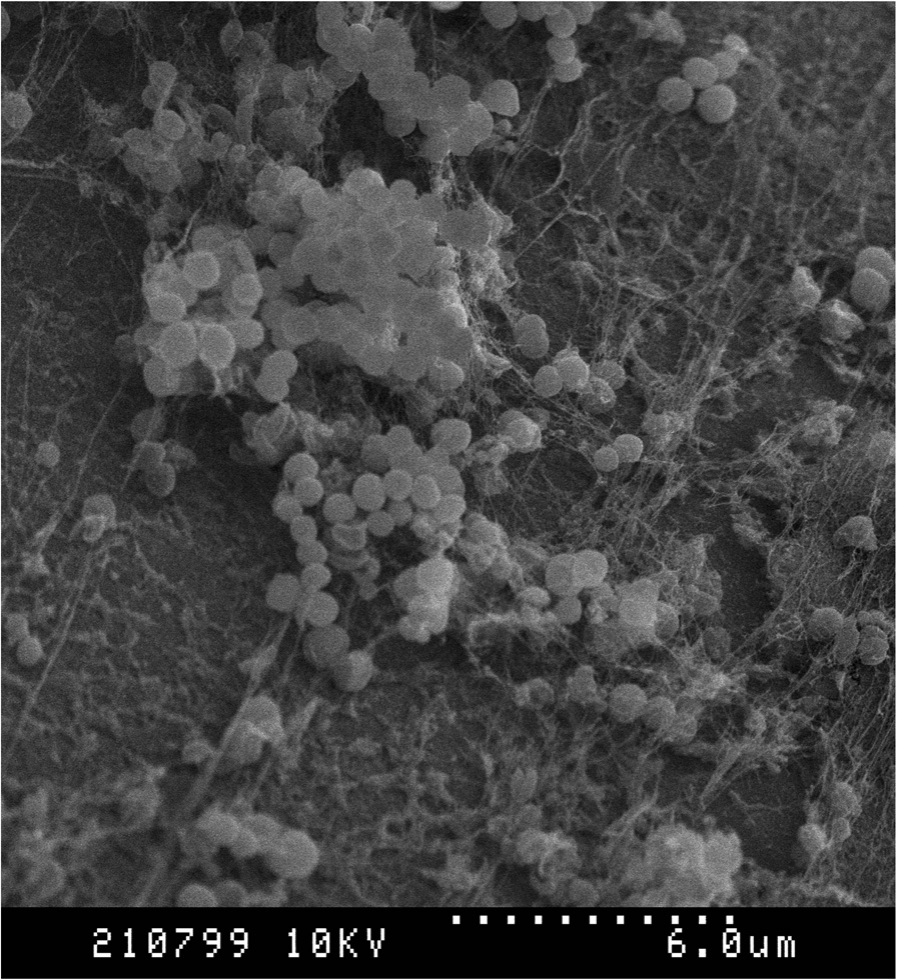
**SEM Images of MRSP biofilm formation on 316L stainless steel orthopedic bone screws.** Dotted line = 6 um. Large aggregates of cocci and irregularly produced extracellular polymeric substance are apparent.

Scanning electron microscopic evaluation of *S. pseudintermedius* biofilm production (Figures 2 and 3) on 316L orthopaedic bone screws revealed the presence of aggregates of cocci in addition to large amounts of irregularly produced EPS.

## Discussion

The majority of *S. pseudintermedius* isolates evaluated in this study were able to produce biofilm, with 96% being classified as either strong or moderate biofilm producers. Biofilm production by *S. pseudintermedius* may play an important role in the pathophysiology of disease and potentially colonization, and could be a contributing factor in the rapid, worldwide emergence of MRSP [6,22]. Biofilm production has been correlated with clinical infection in other *Staphylococcus* spp., and further study into the role of biofilm formation in *S. pseudintermedius* is required [20,21].

Biofilm formation was not different between isolates of MRSP and MSSP, which may correspond to their equivalent virulence clinically [33]; however, the number of MSSP isolates that was studied was low. Regardless of methicillin resistance, biofilm formation may play a role in clinical infection with *S. pseudintermedius*, and further study into the biofilm forming ability of MSSP with a larger number of isolates is warranted.

*S. pseudintermedius* isolates from dogs with clinical infections did not have a significantly different biofilm forming ability compared with isolates from colonized dogs. This is not surprising given the similar distribution of MRSP clones amongst both infection and colonization isolates, and the fact that strains present on an individual (colonization) are presumably the source of infection. Biofilm formation may also be an important virulence factor allowing for colonization of *S. pseudintermedius* in dogs, facilitating survival in the upper respiratory tract and other body sites.

Investigation into the molecular epidemiology of isolates evaluated revealed the predominance of dru clusters 11a and 9a, corresponding to ST68 and ST71. These dru types are also the two most common clones in North America [34]. A recent European study investigated the biofilm forming ability of 23 MRSP isolates and reported significantly greater *in vitro* biofilm production by ST71 compared to other strains [22]. The same was not noted here; however, it must be noted that ST68 was not evaluated in that study. It is possible that both ST68 and ST71 are abundant biofilm producers. This could be a potential reason for why these two clones have emerged internationally as predominant MRSP clones amongst a highly diverse MSSP population [6,23].

The majority of *S. pseudintermedius* isolates contained *ica*A and *icaD*, which is consistent with a study in *S. epidermidis* which showed that isolates producing some level of biofilm using an MPA contained some genes of the *ica* operon [28]. Yet, there was no apparent association between the presence of either gene and biofilm production or methicillin resistance. Biofilm formation in staphylococci is complex and further study into the role of the *ica* operon and other potential genes in *S. pseudintermedius* is warranted.

The SEM images obtained after incubation of a strong biofilm forming isolate of *S. pseudintermedius* revealed biofilm formation on an orthopaedic implant. The biofilm was characterized with several aggregates of bacterial cells along with large amounts of amorphous EPS. This information provides visual evidence that *S. pseudintermedius* can form biofilm rapidly (within 24 hrs) on stainless steel in an *in vitro* setting and is likely the same *in vivo* following placement of a surgical implant.

## Conclusion

The majority of *S. pseudintermedius* isolates evaluated in this study were able to produce biofilm and this may be an important virulence factor in the rapid emergence of this bacterium in veterinary hospitals worldwide. There appears to be no association between biofilm formation and methicillin resistance, infection vs colonization source and clonal complex. As with other staphylococci, *ica* produced PIA may play a role in *S. pseudintermedius* biofilm formation, but the exact mechanism of biofilm formation in *S. pseudintermedius* requires further study.

## Competing interests

The authors declare that they have no competing interests.

## Authors’ contributions

AS and JSW were responsible for study design and manuscript preparation. MW and JR performed microbiological testing. All authors read and approved the final manuscript.
